# Early TICI 2b or Late TICI 3—Is Perfect the Enemy of Good?

**DOI:** 10.1007/s00062-021-01048-8

**Published:** 2021-06-30

**Authors:** F. Flottmann, N. van Horn, M. E. Maros, R. McDonough, M. Deb-Chatterji, A. Alegiani, G. Thomalla, U. Hanning, J. Fiehler, C. Brekenfeld, GSR investigators

**Affiliations:** 1 Department of Diagnostic and Interventional Neuroradiology, University Medical Center Hamburg-Eppendorf, Haus Ost 22 (O 22), Martinistr. 52, 20246 Hamburg, Germany; 2 Department of Neuroradiology, Medical Faculty Mannheim, Heidelberg University, Mannheim, Germany; 3 Department of Biomedical Informatics at the Heinrich-Lanz-Center, Medical Faculty Mannheim, Heidelberg University, Mannheim, Germany; 4 Department of Neurology, University Medical Center Hamburg-Eppendorf, Hamburg, Germany

**Keywords:** Ischemic stroke, Thrombectomy, Endovascular therapy, Retrievals, Prognostic factors

## Abstract

**Background and Purpose:**

A Thrombolysis in Cerebral Infarction (TICI) score of 3 has been established as therapeutic goal in endovascular therapy (EVT) for acute ischemic stroke; however, in the case of early TICI2b reperfusion, the question remains whether to stop the procedure or to continue in the pursuit of perfection (i.e., TICI 2c/3).

**Methods:**

A total of 6635 patients were screened from the German Stroke Registry. Patients who underwent EVT for occlusion of the middle cerebral artery (M1 segment), with final TICI score of 2b/3 were included. Multivariable logistic regression was performed with functional independence (modified Rankin Scale, mRS at day 90 of 0–2) as the dependent variable.

**Results:**

Of 1497 patients, 586 (39.1%) met inclusion criteria with a final TICI score of 2b and 911 (60.9%) with a TICI score of 3. Patients who achieved first-pass TICI3 showed highest odds of functional independence (Odds ratio [OR] 1.71, 95% confidence interval [95% CI] 1.18–2.47). Patients who achieved TICI2b with the second pass (OR 0.53, 95% CI 0.31–0.89) or with three or more passes (OR 0.44, 95% CI 0.27–0.70) had significantly worse clinical outcomes compared to first-pass TICI2b. TICI3 at the second pass was by trend better than first-pass TICI2b (OR 1.55, 95% CI 0.98–2.45), but TICI3 after 3 or more passes (OR 0.93, 95% CI 0.57–1.50) was not significantly different from first-pass TICI2b.

**Conclusion:**

First-pass TICI2b was superior to TICI2b after ≥ 2 retrievals and comparable to TICI3 at ≥ 3 retrievals. The potential benefit in outcome after achieving TICI3 following further retrieval attempts after first-pass TICI2b need to be weighed against the risks.

**Supplementary Information:**

The online version of this article (10.1007/s00062-021-01048-8) contains supplementary material, which is available to authorized users.

## Introduction

Endovascular therapy (EVT) has been established as the standard of care for acute large-vessel occlusion (LVO) in the anterior circulation ischemic stroke [1]. In the past, procedural success of EVT had been defined by a final thrombolysis in cerebral infarction (TICI) score of 2b and 3 [2]; however, several studies have reported improved clinical outcome in patients with a final TICI score of 3 vs. those with a final score of 2b [3–5]. Furthermore, a TICI score of 2c has been defined as nearly complete reperfusion [6]. The clinical importance of achieving at least this level of reperfusion has been illustrated in a recent study by Dargazanli et al., which showed that patients with a TICI score of 2c/3 had a superior outcome compared to those with a TICI score of 2b [7]. As a result, the current ESO/ESMINT guidelines recommend TICI 3 reperfusion to be the therapeutic goal of EVT, if reasonably and safely achievable [8].

In the case of early TICI 2b reperfusion, the practical question remains whether to stop or continue the procedure, with the goal of achieving TICI 3. Barring any potential risks associated with increasing retrieval count, one could perform as many attempts as needed to achieve the TICI 2c/3 reperfusion goal; however, multiple retrieval attempts have been associated with worse clinical outcome following EVT [9–11], as well as with an increased risk of intracranial hemorrhage [12, 13].

For the present study, we included only patients with occlusions of the M1 segment of the MCA from a large multicenter registry, in an effort to rule out differing locations of occlusion as a confounding factor of reperfusion success [14, 15]. Our aim was to compare outcomes of patients with a final TICI score of 3 with a final TICI score of 2b, stratified by the number of retrieval attempts required.

We hypothesized that patients with a final TICI score of 2b during the first attempt have the same or superior odds of good clinical outcome compared to patients who achieve TICI 3 after more than one attempt.

## Methods

### Patient Selection

A total of 6635 patients were initially analyzed from the German Stroke Registry—Endovascular Treatment database (GSR-ET 07/2015–04/2018; ClinicalTrials.gov Identifier: NCT03356392). Patients who were treated between June 2015 and December 2019 were included. The GSR-ET is an ongoing, open-label, prospective, multicenter registry of consecutively recruited EVT patients, with 25 participating stroke centers in Germany [16].

The inclusion criteria were: 1. acute ischemic stroke due to large-vessel occlusion in patients aged ≥ 18 years, 2. decision to perform EVT, 3. occlusion of the M1 segment of the middle cerebral artery (MCA), confirmed on digital subtraction angiography (DSA), and documented location of occlusion, 4. a final TICI score of 2b or 3 following EVT, 5. documented age, admission National Institutes of Health Stroke Scale (NIHSS) score, Alberta stroke program early CT score (ASPECTS), reported number of retrieval attempts in EVT, modified Rankin Scale (mRS) score at 90 days (mRS90), and available information on the administration or withdrawal of intravenous recombinant tissue plasminogen activator (iv-thrombolysis). Patients with spontaneous reperfusion before EVT were excluded from the analysis.

Study protocols and procedures were in accordance with the ethical guidelines of the leading ethics committee with additional approval from the local ethics committees of the participating hospitals. The study was conducted in compliance with the Declaration of Helsinki.

### Data Acquisition and Management

Data acquisition was performed according to the previously described protocol of the GSR-ET [16, 17]. All data were collected by the local neurointerventionalists and neurologists and underwent standardized quality checks to control for consistency, plausibility, and completeness.

The ASPECTS was determined on admission nonenhanced computed tomography (CT) scans or diffusion-weighted imaging (in the case of magnetic resonance imaging, MRI). The TICI score was assessed on the final angiographic series by the treating neurointerventionalist. The number of retrieval attempts was documented following intervention by the neurointerventionalist and included both retrieval attempts with stent retriever devices as well as direct thrombus aspiration attempts. The modified Rankin Scale (mRS) score was assessed at 90 days. Good clinical outcome was defined as an mRS score at day 90 of $\leq$ 2 and was assessed as the primary outcome.

### Statistical Analysis

All analyses were performed with the R statistics program (v. 3.6.3, R Core Team 2019, Vienna, Austria; RStudio IDE v. 1.1.463, Boston, MA, USA) [18]. Normally distributed variables are reported as mean and standard deviation (SD). Non-normally distributed data are reported as median and interquartile range (IQR). Categorical variables are reported as proportions. We performed multivariable logistic regression analysis with good clinical outcome (mRS score at day 90 of $\leq$ 2) as the dependent variable, adjusted for age, NIHSS and ASPECTS on admission, administration of iv thrombolysis, and the location of occlusion (occlusion of the proximal vs. distal M1 segment). The final TICI score and number of retrieval attempts were stratified into TICI 2b or TICI 3 following the first, second or ≥ third retrieval attempt. Figures were created using ggplot2 (R statistics program). *P*-values < 0.05 were considered significant.

## Results

### Baseline Characteristics

**Fig. 1:**
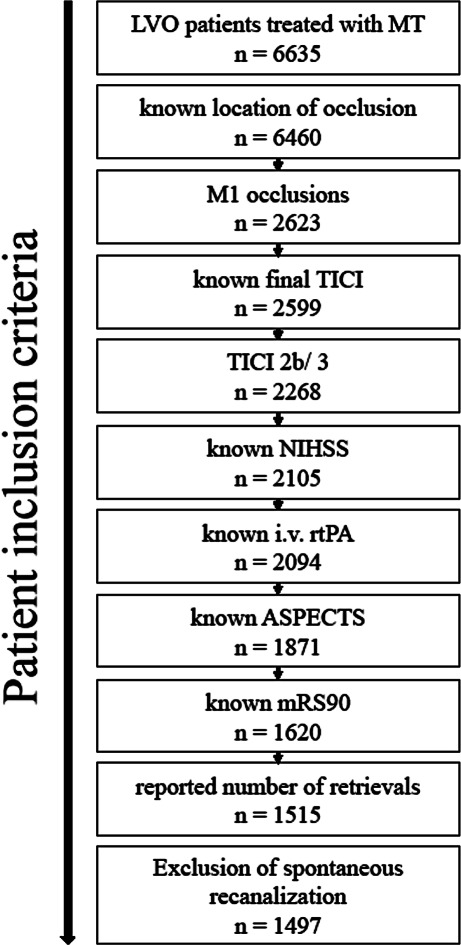
Patient selection flowchart. *LVO* Large Vessel Occlusion, *MT* Mechanical Thrombectomy, *M1* first middle cerebral artery segement, *TICI* Thrombolysis in Cerebral Infarction score, *NIHSS* National Institutes of Health Stroke Scale, *rtPA* recombinant tissue Plasminogen Activator, *ASPECTS* Alberta Stroke Programme Early CT Score, *mRS90* modified Rankin scale at 90 days

Basic characteristics, procedural and clinical outcome are displayed in Table 1. Of 6635 screened patients, 1497 fulfilled the inclusion criteria (Fig. 1). Of these, 808 patients (54.0%) were female. The mean age was 73.6 years (±13.3). The median NIHSS score on admission was 15 [11–18] and the median ASPECTS score on admission imaging was 9 [7–10]. The median number of device passes was 1 [1, 2]. Concomitant stenting of the cervical ICA was performed in 4.6% of patients. The median mRS at day 90 was 3 [1–5]. A good clinical outcome was observed in 629 (42%) patients. Mortality rate at day 90 was 24%. A final TICI score of 3 was observed in 911 patients (60.9%), and the remaining 586 patients had final TICI score of 2b (39.1%).

**Table 1:** Basic characteristics, procedural and clinical outcome

Variable	TICI 2b (*N* = 586)	TICI 3 (*N* = 911)
*Age, years (mean, SD)*	72.8 (13.9)	74.1 (12.9)
*Female sex (%)*	318 (54.3%)	491 (53.9%)
*Hypertension (%)*	437 (74.8%)	703 (77.5%)
*Diabetes mellitus (%)*	124 (21.2%)	192 (21.1%)
*Dyslipidemia (%)*	198 (33.8%)	378 (41.6%)
*Atrial fibrillation (%)*	241 (41.3%)	414 (45.5%)
*Initial NIHSS score (median, Q1–Q3)*	15 [11–18]	15 [10–18]
*Pre-Stroke mRS score (median, Q1–Q3)*	0 [0–1]	0 [0–1]
*Initial ASPECTS (median, Q1–Q3)*	8 [7–10]	9 [8–10]
*Initial occlusion site (%)*		
Left hemisphere	300 (51.3%)	462 (50.8%)
*Location of vessel occlusion (%)*		
M1 proximal	355 (60.6%)	572 (62.8%)
M1 distal	234 (39.9%)	346 (38%)
*Intravenous tPA, n (%)*	317 (54.1%)	464 (50.9%)
*Onset to admission, min (median, Q1–Q3, n* *=* *868 reported values)*	135 [62–201]	105 [56.5–190]
*Stroke etiology*		
Cardioembolism	314 (54%)	526 (58.1%)
Dissection	2 (0.3%)	9 (1%)
Atherosclerosis	115 (19.8)	174 (19.2%)
Other determined etiology	28 (4.8%)	36 (4%)
Unknown etiology	123 (21.1%)	160 (17.7%)
*Onset to groin puncture, min (median, Q1–Q3, n* *=* *861)*	195 [140–260]	175 [126–246]
*Groin puncture to final TICI, min (median, Q1–Q3, n* *=* *1451)*	42 [26–67]	33 [22–52]
*Onset to reperfusion, min (median, Q1–Q3, n* *=* *848)*	253 [188–320]	219 [162–298]
*Number of retrieval attempts (%)*		
1	235 (40.1%)	558 (61.2%)
2	139 (23.7%)	195 (21.4%)
3	85 (14.5%)	99 (10.9%)
4	55 (9.4%)	25 (2.7%)
5	35 (6.0%)	17 (1.9%)
6 or more	37 (6.3%)	17 (1.9%)
*Dissection/Perforation*	10 (1.7%)	15 (1.7%)
*Intracranial hemorrhage (ICH)*	16 (2.7%)	12 (1.3%)
*Modified Rankin Scale (mRS) score at 90 days (median, Q1–Q3)*	4 [2–6]	3 [1–5]
*Mortality (mRS = 6)*	109 (23%)	145 (18.1%)
*Functional independence (mRS ≤ 2)*	192 (32.8%)	438 (48.1%)

*NIHSS* National Institutes of Health Stroke Scale; *mRS*  modified Rankin scale; *ASPECTS* Alberta Stroke Programme Early CT Score; *M1* first middle cerebral artery segement; *M2* second middle cerebral artery segment; *tPA* tissue Plasminogen Activator; *TICI* Thrombolysis in Cerebral Infarction score

### Proportion of TICI 3 Reperfusions and Good Clinical Outcome by Retrieval Attempt

**Fig. 2:**
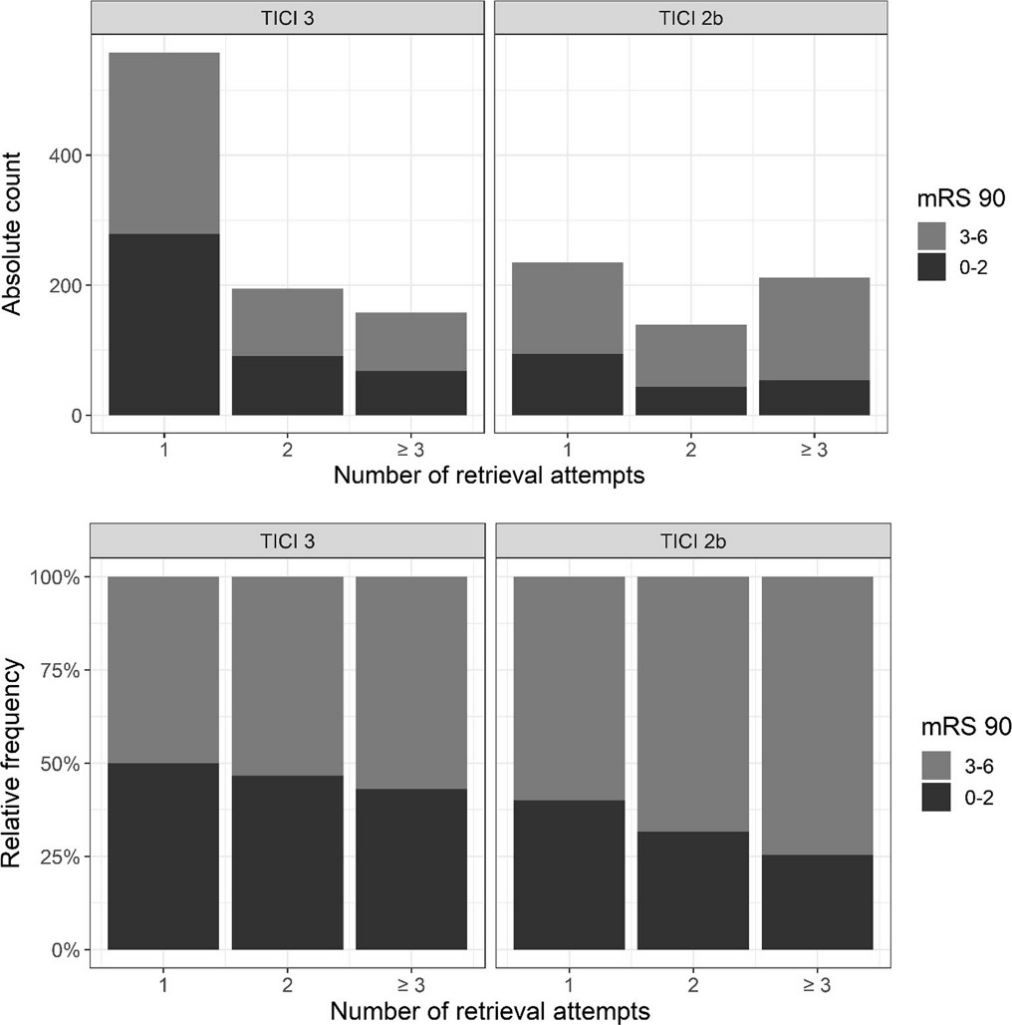
Rate of good clinical outcome (defined as mRS of 0–2 at day 90) by final TICI score and number of retrieval attempts. *mRS* *90* modified Rankin scale at day 90, *TICI* thrombolysis in cerebral iInfarction

Of those patients who only underwent 1 retrieval attempt, a final TICI score of 3 was observed in 558 (70%) patients (Table 2). Of those who underwent 4 or more retrieval attempts, the rate of a final TICI 3 score declined to 31.3–32.6%. Fig. 2 depicts the rate of good clinical outcome by final TICI score and the total number of retrieval attempts performed. Of 558 patients with first pass TICI 3, 279 (50%) achieved a good clinical outcome. Of 235 patients with first pass TICI 2b, 94 (40%) achieved a good clinical outcome.

**Table 2:** TICI grade and clinical outcome by number of retrievals

Number of retrievals	1 pass (*n* = 793)		2 passes (*n* = 334)		≥ 3 passes (*n* = 370)	
Final TICI	TICI 2b	TICI 3	TICI 2b	TICI 3	TICI 2b	TICI 3
*n*	235	558	139	195	212	158
% per pass	29.7%	70.3%	41.6%	58.4%	57.3%	42.7%
Functional independence *n*, (%)	94 (40)	279 (50)	44 (32)	91 (47)	54 (26)	68 (43)
Mortality *n*, (%)	53 (23)	118 (21)	40 (29)	43 (22)	78 (37)	33 (21)

*TICI* Thrombolysis in Cerebral Infarction score

### Multivariable Analysis

The multivariable logistic regression analysis (adjusted for predictors, Table 3) revealed a negative association of older age (per-year increase OR 0.93, 95% CI 0.92–0.94, *p* < 2 × 10^−16^) and higher NIHSS score on admission (per-point increase OR 0.89, 95% CI 0.87–0.91, *p* < 2 × 10^−16^) with good clinical outcome. Furthermore, distal M1 occlusions were associated with a better outcome compared to proximal M1 occlusions (OR 1.54, 95% CI 1.19–1.98, *p* = 0.0008), as was iv-thrombolysis (OR 1.76, 95% CI 1.37–2.26, *p* = 8.72 × 10^−6^), and an ASPECTS of 10 (OR 2.42, 95% CI 1.46–4.10, *p* = 0.0007) or 8–9 (OR 2.07, 95% CI 1.26–3.47, *p* = 0.0047).

**Table 3:** Summary table of the logistic regression model with good clinical outcome (mRS at day 90 of 0–2) as the dependent variable

Predictor variable	OR (95% CI)	Coefficient^b^	*P*-value
Age^a^	0.93 (0.92–0.94)	−0.07	< 2 × 10^−16^
NIHSS^a^	0.89 (0.87–0.91)	−0.12	< 2 × 10^−16^
ASPECTS 0–5	–	Ref	–
ASPECTS 6–7	1.43 (0.83–2.49)	0.36	0.20
ASPECTS 8–9	2.07 (1.26–3.47)	0.73	0.0047
ASPECTS 10	2.42 (1.46–4.10)	0.89	0.0007
Proximal M1 occlusion	–	Ref.-	–
Distal M1 occlusion	1.54 (1.19–1.98)	0.43	0.0008
Iv-Thrombolysis	1.76 (1.37–2.26)	0.57	8.72 × 10^−6^
TICI 2b at first pass	–	Ref.-	–
TICI 3 at first pass	1.71 (1.18–2.47)	0.53	0.0046
TICI 2b at 2nd pass	0.53 (0.31–0.89)	−0.63	0.017
TICI 3 at 2nd pass	1.55 (0.98–2.45)	0.44	0.06
TICI 2b at 3rd or more passes	0.44 (0.27–0.70)	−0.82	0.0006
TICI 3 at 3rd or more passes	0.93 (0.57–1.50)	−0.07	0.76
(Intercept)	94.26 (36.36–249.61)	4.55	< 2 × 10^−16^

*NIHSS* National Institutes of Health Stroke Scale, *ASPECTS* Alberta stroke programme early CT score, *TICI* Thrombolysis in Cerebral Infarction score

^a^Age and NIHSS were treated as continuous variables; ASPECTS and TICI at the n^th^ retrieval were treated as factors

^b^Coefficients are reported on the logit scale

**Fig. 3:**
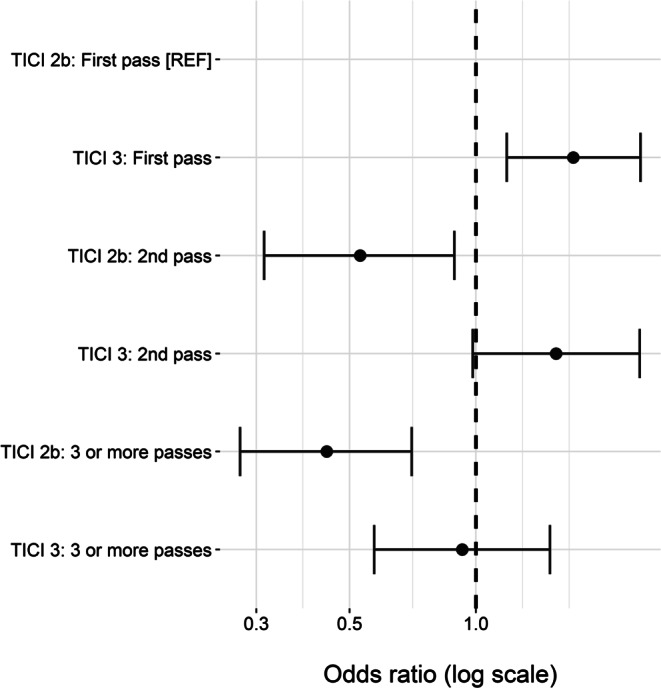
Odds ratio of good clinical outcome (defined as mRS of 0–2 at day 90) by final TICI score and number of retrieval attempts, adjusted for age, NIHSS score on admission, location of occlusion (proximal vs. distal segment of the MCA), ASPECTS on admission and iv-thrombolysis. *mRS* modified Rankin scale, *TICI* thrombolysis in cerebral infarction, *MCA* middle cerebral artery, *ASPECTS* Alberta stroke programme early CT score

First-pass TICI 2b patients comprised the reference group (Fig. 3). Patients with first-pass TICI 3 reperfusion had the highest odds of a good clinical outcome (OR 1.71, 95% CI 1.18–2.47, *p* = 0.0046). Second-pass TICI 2b had a significantly worse clinical outcome than first-pass TICI 2b (OR 0.53, 95% CI 0.31–0.89, *p* = 0.017), whereas second-pass TICI 3 was by trend better than first-pass TICI 2b with a borderline result (OR 1.55, 95% CI 0.98–2.45, *p* = 0.06). When examining patients who underwent ≥ 3 retrieval attempts, it was found that those who thus achieved TICI 2b had a significantly worse clinical outcome compared to first-pass TICI 2b (OR 0.44, 95% CI 0.27–0.70, *p* = 0.0006), whereas those who thus achieved TICI 3 had outcomes comparable to first-pass TICI 2b (OR 0.93, 95% CI 0.57–1.50, *p* = 0.76).

## Discussion

In the present study, we observed two effects: first, a final TICI score of 3 outperformed a final TICI score of 2b within each group of patients who underwent a certain number of retrieval attempts. Second, both TICI 3 and TICI 2b showed decreasing odds of good clinical outcome with increasing number of retrieval attempts. Hence, patients with late TICI 3 reperfusion (after 3 or more retrievals) and patients with early TICI 2b reperfusion (after the first retrieval) showed comparable odds of good clinical outcome. Furthermore, the rate of TICI 3 was ~70% in patients who underwent 1 attempt and decreased to ~30% for patients who underwent 4 or more retrieval attempts.

In our patient cohort, the proportion of TICI 3 amongst all patients with sufficient reperfusion (TICI 2b–3) was 61%, which is slightly higher than in the ESCAPE-NA 1 trial control group (259/480 patients, 54%) [19]. The difference could be explained by the fact that ESCAPE-NA 1 had a central imaging core laboratory, while our registry used self-reported final TICI scores, which are potentially subject to bias [17]. Furthermore, we only included occlusions of the M1 segment of the MCA, which showed higher rates of reperfusion than occlusions of the ICA [15].

An improved clinical outcome after TICI 3 vs. TICI 2b reperfusion has been previously described [3, 4], as has decreased infarct growth after TICI 3 reperfusion [20]. Dargazanli et al. reported rates of functional independence of 71.7% vs. 50.5% for TICI 3 vs. TICI 2b [4], while Chamorro et al. reported rates of functional independence of 67.6% vs. 56.8% [20]. In this study, we observed rates of 48.1% vs. 32.8% for TICI 3 and 2b, respectively. Furthermore, as previously described [22], patients treated with intravenous thrombolysis (rtPA) prior to mechanical thrombectomy were more likely to achieve higher reperfusion rates and subsequently favorable outcome as demonstrated in Table 1, [21] while a recently conducted meta-analysis pooling 20 studies with more than 5000 patients conducted by Kaesmacher et al. favored neither treatment approach [22]. The overall comparatively lower rates of good clinical outcome in our GSR-ET cohort have been previously described and discussed [17]. Despite this, we found a similar reduction in the rate of good clinical outcome in patients with TICI 2b compared to TICI 3.

In a large multicenter cohort of patients with M1 occlusions, we observed the highest odds of good clinical outcome in patients with TICI 3 reperfusion following a single retrieval, confirming the previously described first-pass effect [23, 24]. Furthermore, when comparing patients who underwent a certain number of retrieval attempts, we found higher odds of good clinical outcome after TICI 3 reperfusion compared to TICI 2b. García-Tornel et al. analyzed the association of final TICI score and number of retrieval attempts in detail [25]. They also observed an overall superior clinical outcome after TICI 3 compared to TICI 2b. In contrast to our findings, they reported comparable rates of good clinical outcome in patients who achieved TICI 3 during the first three passes, with declining odds only being observed following the fourth retrieval attempt. On the other hand, they reported a negative impact of each retrieval attempt on outcome, beginning with the second pass in patients who achieved TICI 2b, which we were able to confirm; however, this part of their analysis was not adjusted for confounders and included a comparatively small number of patients. In our study the odds for a good clinical outcome decreased with the second pass in patients with TICI 2b reperfusion and with the third pass in patients with TICI 3 reperfusion. Due to this effect, the odds of good clinical outcome were comparable between late TICI 3 and early TICI 2b reperfusion.

Our analysis suffers from an important limitation. García-Tornel et al. described the phenomenon of sudden recanalization, defined as a variation of TICI score from 0–1 to 2b–3 in a single pass, and demonstrated that sudden recanalization is associated with superior clinical outcome, even when achieved after multiple retrieval attempts [26]. In the present study recanalization success of each retrieval attempt could not be evaluated due to limitations of data collection, and we therefore cannot differentiate between sudden and stepwise recanalization. Perhaps most importantly, we were unable to further differentiate if patients with late TICI 2b/3 had TICI 0–2a or TICI 2b during the first retrieval attempts. Furthermore, due to the retrospective design of this data collection, TICI 2c was not evaluated separately but is included in the TICI 2b classification.

Other limitations are as follows: the number of retrieval attempts, as well as the final TICI score, was reported by the treating neurointerventionalist and was not validated by a central imaging core laboratory [6]; the technical approaches differed according to the local standard of clinical routine at the different centers and a substantial proportion of patients (1108 of 2623 patients with M1 occlusions, 42.2%) had to be excluded from the analysis due to missing data.

From a practical point of view, the most important question is whether or not to continue with mechanical thrombectomy in the case of TICI 2b reperfusion, especially after the first pass. One could argue that if late TICI 3 is as good as early TICI 2b, the former should always be strived for; however, we describe declining rates of TICI 3 reperfusion with subsequent attempts, as has also been previously reported [26]. Furthermore, while TICI 3 after the second pass showed a tendency towards better outcome compared to first-pass TICI 2b (marginally missing the significance threshold), TICI 3 after 3 or more passes was comparable to first-pass TICI 2b. In addition, with each further retrieval attempt, the patient has an increasing risk of a late TICI 2b result, which was associated with inferior clinical outcome compared to first-pass TICI 2b. Hence, perfect could indeed be the enemy of good. However, due to the beforementioned limitations, a thorough documentation of TICI grade after each retrieval attempt in a large patient cohort would be needed to investigate whether or not there is a significant benefit of further thrombectomy attempts after first-pass TICI 2b has been achieved. Albeit technically challenging, a randomized controlled trial would be needed to ultimately clarify this question.

Our data suggest that each additional retrieval attempt has to be undertaken with caution, taking into account a variety of factors, including the size of the perfusion deficit, eligibility of iv thrombolysis, and the location of the persisting occlusion [27].

## Conclusion

First-pass TICI 2b was superior to TICI 2b after ≥ 2 retrievals and comparable to TICI 3 at ≥ 3 retrievals. Therefore, the negative effects of further retrieval attempts, such as declining odds of good outcome in late TICI 2b, have to be considered.

## Coinvestigators

List of all coinvestigators of the German Stroke Registry Steering Committee was blinded for review.

## Supplementary Information

Fig. 4: Rate of TICI 3 reperfusions stratified by total of retrieval attempts (*dark gray:* final TICI score of 3, *light grey:* final TICI score of 2b)
