# Supplementary material for: Early TICI 2b or Late TICI 3—Is Perfect the Enemy of Good?
Source: Clin Neuroradiol. 2021 Jun 30;32(2):353–60. doi: 10.1007/s00062-021-01048-8 (PMC9187567; doi:10.1007/s00062-021-01048-8)
Supplement: Supplementary file 1 — Fig. 4: Rate of TICI 3 reperfusions stratified by total of retrieval attempts (dark gray: final TICI score of 3, light grey: final TICI score of 2b) [file 62_2021_1048_MOESM1_ESM.docx]

Supplemental material

Figures:


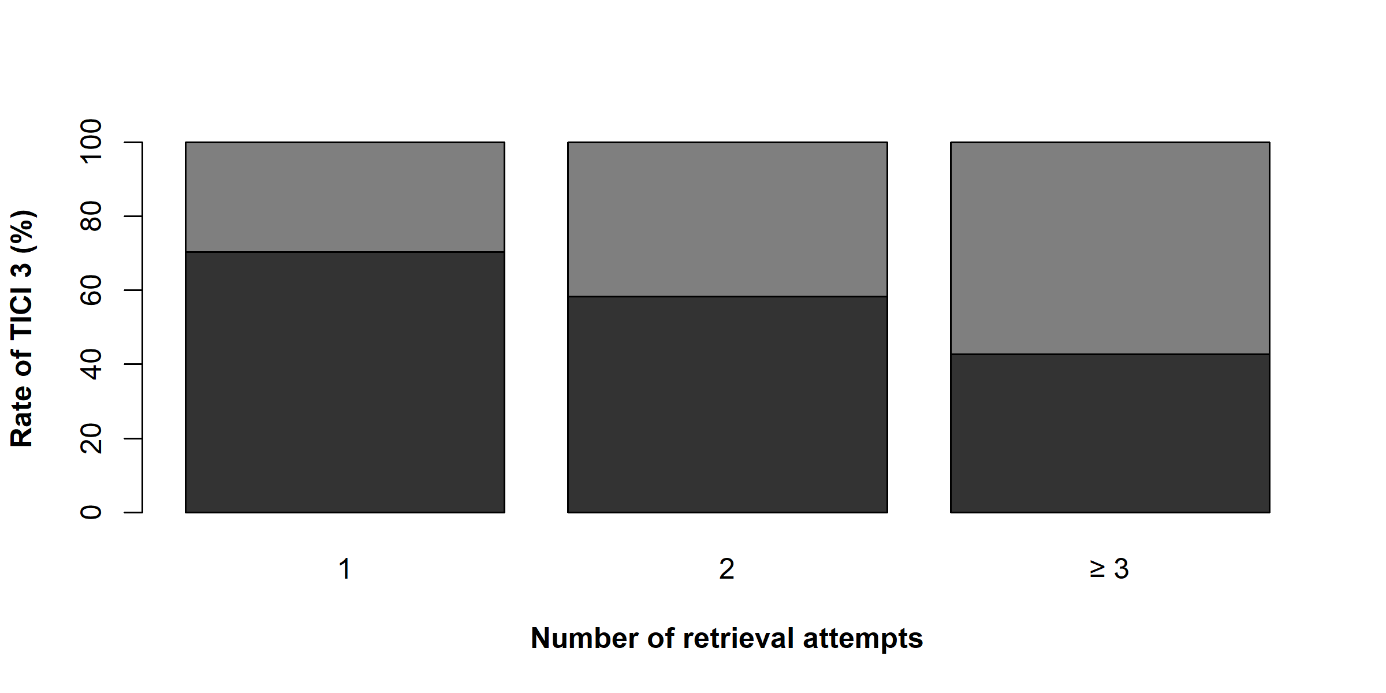
Figure 4: Rate of TICI 3 reperfusions stratified by total of retrieval attempts (dark gray: final TICI score of 3, light grey: final TICI score of 2b).
